# P04.47. Parents’ satisfaction in a department of integrative pediatric oncology: a ten year experience

**DOI:** 10.1186/1472-6882-12-S1-P317

**Published:** 2012-06-12

**Authors:** A Laengler, R Seiler, A Buessing, T Ostermann, G Kameda, P Heusser

**Affiliations:** 1 Centre for Integrative Pediatric Oncology Gemeinschaftskrankenhaus, Herdecke, Germany; 2 Witten Herdecke University, Witten, Germany

## Purpose

In the Gemeinschaftskrankenhaus Herdecke (GKH) children and adolescents with cancer are routinely treated with integrative medicine approaches. In addition to guideline-based conventional treatment a multimodal therapy concept is applied which includes anthroposophic pharmacotherapy (particularly mistletoe extracts) and non-pharmacologic therapies (e.g. art-therapies, external embrocations, eurythmy therapy). We analysed the recalled satisfaction of parents of children treated for cancer in the centre for integrative pediatric oncology at GKH from 1999 to 2008.

## Methods

The parents were anonymously asked to report their satisfaction with treatment and outcome as well as additional items addressing aspects of integrative treatment, using the modified CSQ-8 questionnaire, a validated screening tool for detecting patient satisfaction. Descriptive statistics were gathered.

## Results

Of the 98 mailed questionnaires 68 were returned (69%). Mistletoe extracts were used in 57 children (84%); they were tolerated well by 51 children (90%), while 23% of the parents reported side effects. In February 2010, 54 of the 68 responders (79%) stated a good health status, while 13 children (19%) had died of their tumour; 1 child (1.5%) was still in therapy. Treatment benefit was stated as very high by 58 of the parents (85%). Sixty-seven percent described the quality of care as excellent; 65% said that their needs were met in most areas. Seventy-two percent were very satisfied with the extent of help; 88% would come back if their child needed further help;, 85% were very satisfied with the treatment on the whole; and 82.5% stated that their child had received the kind of treatment they wanted. The mean of the overall impression on a scale of 1-10 was very high (9.3).

## Conclusion

There was a high level of parent satisfaction, indicating that the integrative concept was accepted well by the responding parents.

